# Understanding the Impact of Electronic Medical Record Use on Practice-Based Population Health Management: A Mixed-Method Study

**DOI:** 10.2196/medinform.4577

**Published:** 2016-04-04

**Authors:** Isaac Vaghefi, John B Hughes, Susan Law, Michel Lortie, Chad Leaver, Liette Lapointe

**Affiliations:** ^1^ School of Management State University of New York at Binghamton Binghamton, NY United States; ^2^ Faculty of Medicine McGill University Montreal, QC Canada; ^3^ St Mary’s Research Centre Montréal, QC Canada; ^4^ Canada Health Infoway Montréal, QC Canada; ^5^ Desautels Faculty of Management McGill University Montreal, QC Canada

**Keywords:** primary health care, electronic medical records, population health management, medical informatics, practice-based care

## Abstract

**Background:**

Practice-based population health (PBPH) management is the proactive management of patients by their primary care clinical team. The ability of clinics to engage in PBPH and the means by which they incorporate it in a clinical setting remain unknown.

**Objective:**

We conducted the Canadian Population Health Management Challenge to determine the capacity and preparedness of primary care settings to engage in PBPH using their existing medical record systems and to understand the complexities that may exist in PBPH implementation.

**Methods:**

We recruited a sample of electronic medical record (EMR) -enabled and paper-based clinics from across Canada to participate in the challenge. The challenge required clinic staff and physicians to complete time-controlled, evidence-based practice reviews of their patients who may benefit from evidence-informed care, treatment, or interventions across five different areas (immunization, postmyocardial infarction care, cancer screening, diabetes management, and medication recall). We formulated a preparedness index to measure the capacity of clinics to engage in PBPH management. Finally, we conducted follow-up qualitative interviews to provide richer understanding of PBPH implementation and related issues (ie, challenges and facilitators).

**Results:**

A total of 11 primary care clinics participated, representing 21 clinician practices. EMR-enabled clinics completed a full review of charts in an average of 1.37 hours. On the contrary, paper-based clinics reviewed nearly 10% of their charts in an average of 3.9 hours, hinting that they would have required an estimated 40 hours to complete a review of charts in their practice. Furthermore, the index revealed a major gap in preparedness between the EMR and paper-based clinics (0.86–3.78 vs 0.05–0.12), as well as a broad range among the EMR clinics. Finally, building on the results of the qualitative analysis, we identified factors facilitating the integration of PBPH.

**Conclusions:**

Our results suggest that EMR usage is pivotal in setting the foundation to support PBPH. The wide range of performance variation among EMR-enabled clinics suggests that EMR functionality and optimization, its support of clinical practice workflow, and policy issues to ensure adoption of standards are critical issues to facilitate PBPH.

## Introduction

### Context

In Canada, the federal government spends 50% of its total budget on the Canada Health Transfer to the provinces and territories [1]. One common goal across jurisdictions in recent years has been to improve and transform primary care [2,3]. Statistics show that four types of chronic diseases (cardiovascular disease, cancers, chronic obstructive pulmonary disease, and diabetes) are the major causes of hospitalization in Canada and are responsible for significant mortality (153,000 patients or 75% of all deaths) and could benefit from improved primary care prevention [4]. Provincially, providing care for patients with complex chronic conditions (largely older adults) and mental health issues accounts for near 50% of health care expenditures in Ontario, Canada’s most populous province [5]. All of the above conditions involve a significant role for community-based primary care providers from the aspects both of care and treatment of these conditions and of prevention among at-risk segments of their panel of patients. To prevent the negative impacts of such diseases and decrease their associated costs, it is important that regular care providers be able to proactively identify vulnerable or at-risk patients who may benefit from screening, treatment, or interventions [6-8].

To do this, scholars and practitioners have increasingly recognized the importance of actively managing population health at the primary care level as an essential factor in improving quality of care outcomes [6,9]. It is widely recognized that, to provide a better quality of care for patients with acute and chronic diseases and improve health outcomes, it is important to effectively prevent disease and disability at a population or community level, and potentially at the district or country level [10-12]. This issue is becoming especially important, due to the challenges presented to public health in the international primary care context by the upsurge of new diseases and infections, immigration and change in community demographics, social and economic determinants of health, and enduring environmental disasters [3].

One major effort has been to establish practice-based population health (PBPH) methodologies and procedures for primary care practice [13,14]. PBPH management has been defined as “an approach to care that uses information on a group (population) of patients within a primary care practice or group of practices (practice based) to improve the care and clinical outcomes of patients within that practice” [15]. PBPH focuses on an entire population or its subset (eg, a community) with a common health problem or risk exposure. The goal is to identify and address everyone who is within the target population, and to pinpoint health priorities and actions through a systematic assessment and selection process, with an emphasis on provision of equitable prevention services [16]. To ensure that preventive maneuvers are updated and to fully implement PBPH management, clinical teams need accurate data on the population from their medical records [13,17]. However, medical records kept in paper format make it difficult to optimally retrieve documented information and subsequently integrate PBPH into daily practice workflow [18,19].

One of the key elements that can improve practice engagement with PBPH is the integration of information technology, data quality of electronic patient records, and integrated administrative and clinical workflow [7,20]. This is generally conducted through implementation, adoption, and use of electronic medical record (EMR) or electronic health record (EHR) systems [17,18,21]. Indeed, previous research has shown that the adoption of technological advances such as EMR and EHR systems is key to enabling positive outcomes from implementing PBPH [15,19].

The benefits of electronic systems have been well recognized in the extant literature. For instance, research shows that the use of EMRs in hospital and ambulatory care settings can improve patient safety and reduce adverse events, by using alerts and reminders [22]. In addition, use of EMR functionalities and data quality management with clinicians can assist in improving preventive care maneuvers and chronic disease management [19]. Furthermore, EMR use has been found to lower the cost of care [23] by reducing staff time required for paper-based administrative duties and smoothing the clinic’s management workflow for laboratory results [24]. Nevertheless, other existing studies examining the benefits of EMR implementation have provided mixed support for these areas of value [19,22,25,26].

In the context of population health management and PBPH, clinicians’ use of and consultation with patient data and the provision of alert and reminder functionalities has been shown to support chronic disease management [27]. Use of EMR data in this regard is independent of electronically enabled chronic disease management software or programs that may function separately from the EMR. Indeed, use of EMR data for PBPH also depends on several factors, such as technical feasibility to access individual-level or aggregated EMR data reports and clinicians’ capacity to perform aggregated review and execute follow-up with identified patients. Nevertheless, previous research has highlighted five main approaches in which use of EMR data can support PBPH [15]. First, clinicians can use EMRs to effectively identify the communities of patients who need additional health care services. For instance, lists can be generated of patients who need checkups and follow-up support, or those who require risk-reduction consultation based on specific clinical or demographic indicators. Second, EMRs with functionalities to create reminders or alerts support physicians in conducting follow-up tests, procedures, or education with a patient either within or outside of individual patient encounters. Third, EMR systems may have the ability to send unique notifications based on clinical indicators. Fourth, EMRs can graphically illustrate over time the impact of treatment or preventive maneuvers on longitudinal presentation of clinical laboratory tests or other measured outcomes. EMRs can also generate various quality reports that compare and contrast the practices of caregivers with local (clinicians within the practice), national, or global standards, provide timely access to guidelines on common diagnosis and treatment care plans, and apply quality measures to PBPH management. Fifth, EMRs can display data in various forms (bar charts, tables), or export and print it in different forms, so that users can use data for further analysis [15,27].

Despite the potential benefits, optimizing use of EMR functionalities in primary care has been particularly complex and challenging [28]. In fact, an international survey of 5000 primary care physicians revealed that the adoption and the extent of optimized EMR usage by clinicians in North America is lower than expected [29]. Specifically in Canada, approximately one-quarter of primary care practices still used paper-only records in 2015, with substantial variation in EMR adoption between provinces [30]. Canadian EMR-enabled primary care practices are also ranked below the international average for preforming specific population health management practices [30]. In 2012, at the time of this study, only 18% of primary care physicians in Canada reported improved management and diagnosis of chronic diseases via EMR use; the rate was even lower (3%) for primary care physicians who reported using multifunctionality of their EMR system to support chronic disease management and preventive care among their panel of patients [24]. Therefore, our study sought to understand how clinics can perform PBPH efficiently in the new context enabled by technology.

### Objectives

We report the design and results of the Canadian Population Health Management Challenge, in which we assessed paper-based and EMR-enabled primary care clinics located in Canada on their capacity and preparedness to engage in PBPH management. More specifically, we aimed to answer these questions: How prepared are clinics to adopt PBPH? What are the factors that facilitate PBPH management?

## Methods

### Sampling

We invited a sample of primary care clinics from across Canada to participate in the Population Health Management Challenge. The challenge required clinic staff or a lead physician to complete time-controlled, evidence-based practice reviews of their patients who may benefit from evidence-informed care, treatment, or interventions across five clinical areas (immunization, postmyocardial infarction care, cancer screening, diabetes management, and medication recall). We sought practices with EMR systems and practices with paper-based patient records. Requests for participation were disseminated broadly via Canada Health Infoway’s provincial peer network programs and across provincial EMR funding programs. Programs were encouraged to share the invitation broadly across their networks; therefore, we do not know the total number of invitations disseminated. Community-based primary care clinics or clinician practices were eligible to participate. Clinics interested in participating contacted the study coordinator by email and were later interviewed to determine eligibility and review participation requirements. Clinics that volunteered to participate were required to appoint a lead physician or a staff member for a 6-hour period to complete the challenge at a specified date and time. Real-time monitoring and support was provided while participants completed the time-controlled, evidence-based practice reviews that made up the 6 challenge modules using a Web-based tool. The Web-based tool systematically captured the time to complete each evidence-based review module. All participants completed a Web-based orientation and registration session before the date and time they initiated the challenge. Participants completed 2 rounds of the challenge (round 1 in August 2011 and round 2 in October–November 2011).

### Instruments and Measures

Clinic and clinician practice demographic data were collected during the orientation and training session: (1) the descriptive characteristics of the clinic and participating clinician practices (number of active patients, number of clinicians and care staff, year of graduation), (2) the type and use of chart recording systems (EMR, paper), and (3) the challenge participant’s function within the clinic. The challenge consisted of 6 evidence-based review modules requiring participants to review active patient charts or records and enter the results of their review—all within a specified time limit (please see Multimedia Appendix 1 for formatted screen examples used to capture clinic characteristics and challenge modules).

The challenge modules were designed by a committee of practicing Canadian family physicians and primary care researchers and consisted of multiple indicators to support the appropriate definition of eligible patients within a participating practice who may benefit from evidence-informed care, treatment, or interventions across five focus areas (noted above), each completed sequentially (Table 1). We chose clinical scenarios to represent typical information retrieval situations commonly found in primary care and specifically grounded in current evidence-based practice [21,31-36]. The first task of each module required initial consultation of all patient charts (of the participating physician’s practice) to identify the target patient population that met the selection criterion for the evidence-based review. Beyond a simple registry, all subsequent indicators or tasks within the module were focused on this target population to further define the patients eligible to receive the evidence-based directed care, treatment, or intervention. Finally, each module required participants to specify the source or method of data abstraction (EMR or paper charts), percentage of eligible charts that were actually reviewed within the recorded time, and the degree of confidence (assessed on a 5-point Likert scale) that the abstracted results for each module had captured all eligible patients within the practice.

**Table 1:** Modules and allotted time to complete each one in the Canadian Population Health Management Challenge.

Module	Description	Time limit (minutes)
1	Identify all active patients over the age of 65 years and indicate those who have not received a vaccination against pneumococcal pneumonia.	45
2	Identify all active patients who have had a myocardial infarct and indicate those for whom a statin medication has not been prescribed.	45
3	Prepare a registry, including phone numbers, of all active patients who are female over the age of 50 years and identify those who have not had a mammogram in the last 3 years.	60
4	Prepare a registry, including contact information, of all active patients who are taking the drug metformin and have a creatinine result greater than 150 μmol/L. With the registry in hand, assess the practice’s ability to perform a recall of this medication.	45
5	Identify all active patients diagnosed with type 2 diabetes and indicate those for whom the latest hemoglobin A_1c_test indicates a value greater than 0.070.	60
6	Prepare a registry, including contact information, of all active patients who are taking the drug Avandia and have been diagnosed with congestive heart failure.	45

Participants were allowed 45 to 60 minutes to complete each module. The time taken by each participant was systematically recorded by the Web-based data entry tool with automated time-out features for each module. In the event of a participant time-out, the module was halted (data entry no longer possible) and the data collected to that point were recorded to the database.

To support and enhance our understanding of user experience with the tool and ensure quality of the data collected, we conducted on-site observations at 2 clinics. Each challenge participant completed a follow-up semistructured phone interview. We developed the interview guide based on a review of the extant literature and the observational site visits that we conducted with the first paper-based and EMR-enabled sites while they completed the challenge. The guide was refined jointly with the research team and validated through 2 pilot interviews. All interviews were recorded and transcribed verbatim. As a quality improvement study, this study was not reviewed by a research ethics board. The research team did not consult patients’ records and challenge modules did not require the capture of personal health information.

### Development of the PBPH Preparedness Score

We formulated a preparedness score as a relative measure of a clinic’s capacity to engage in PBPH management. We based clinic preparedness on two key principles: timeliness and completeness. A clinic that requires less time to specify a defined patient population with complete clinical criteria across its full panel of patients is deemed to be more prepared for PBPH management than a clinic that takes longer to complete a full panel review or has incomplete clinical criteria.

We used 2 sets of values to compute the PBPH preparedness score: (1) the total time required to complete the challenge modules, and (2) the percentage of data fields that were completed within challenge modules (degradation factor). We computed the score for each clinic that undertook the challenge on behalf of one or more clinician practices and that was supported by the time data recorded automatically by the Web-based tool and the self-reported proportion of charts actually reviewed to the overall number of patients in the physician’s practice.

For each practice, we computed the mean percentage of modules completed, inclusive of all the data fields in each of the 6 modules. We then defined the mean percentage complete for clinics with multiple participating practices as the average of the mean percentage complete across all the physician practices of the clinic on a module-by-module basis. Then we combined the mean percentage complete for the clinic with the time allocated for the completion of each module and the actual time taken by the clinic to complete the modules for all practices, according to the following formula: score = (mean percentage for clinic × time allocated to complete section) / time taken by all practices to complete section.

The PBPH preparedness score can be interpreted as the percentage of the challenge that the clinic was able to complete in the allotted time. The inverse of the score, multiplied by the overall time allowed to complete the challenge, provides an estimation of the time the clinic would require to complete all tasks across modules that composed the challenge.

More precisely, we defined the percentage complete as the ratio of the number of charts that had all criteria identified (either met the criteria or did not) over the total number of charts in the clinic. This is estimated and self-reported by challenge participants for each module before advancing to the next module whether they reached the time limit or not. For the first participating clinics, we assigned an average percentage complete based on notes taken by the on-site observers. Missing values for the percentage complete of a task were imputed using the following two rules. First, if the practice had complete clinical criteria data fields within the module, we assigned full percentage (100%) complete, as all data elements were present for the required analysis. Second, if such information was not provided, we assigned zero percentage to the associated percentage completed.

We ranked clinics based on their fastest time and completeness of clinical criteria, ordering them from the highest to lowest capacity to conduct PBPH management based on the preparedness score.

We analyzed qualitative data in 2 stages. We first performed a within-case analysis of the resulting transcripts. Within-case analysis allowed us to focus on the particularities of each case. We used documentation and observational data to corroborate and validate the insight provided by the interviews [37]. We then proceeded to a cross-case analysis in order to contrast and compare data and to allow for common patterns to emerge. For the cross-case analysis, we followed a grounded theory approach [38]. Following a round of open coding, we used an axial coding strategy, and we grouped codes with the same content and meaning into categories. From these we identified the following categories: (1) motivation to participate in the challenge, (2) current patient and clinical data retrieval challenges, (3) key learning points, and (4) future developments. Then, through selective coding, we analyzed the patterns.

## Results

A total of 55 clinics responded to the national communications strategy inviting participation in the study. The study coordinator contacted and interviewed interested practices to determine eligibility and review participation requirements. Of these, 11 (8 EMR-enabled; 3 paper-based) clinics volunteered to participate in the challenge. The remaining 44 declined to participate due to lack of time and available staff, or because of personal, business, or operational conflicts with the timing of the data collection periods. Among EMR-enabled clinics, the lack of knowledge about data retrieval and getting queries from an EMR was consistently mentioned as the key reason for nonparticipation. For clinics with paper-based record systems, the task of data retrieval through manual chart reviews was the key barrier to participation. During the orientation session conducted preceding the challenge, 1 paper-based clinic withdrew because the tasks were deemed beyond the capacity of the designated staff member assigned to the challenge.

Among volunteering clinics, the main motivation to participate in the challenge was, first, to assess their performance vis-à-vis other clinics and, second, to evaluate the efficiency of their current practices. Additionally, clinic managers hoped that the challenge could advance adoption and optimized use of EMRs in Canada more generally by highlighting the potential benefits of advanced use to colleagues, regional partners, and government agencies.

Challenge modules were completed either by a primary care physician (4 clinics) or by medical record or information technology staff (7). Table 2 lists the participating clinics and other demographic information such as their location, the practice size and type of medical record keeping system, and the person responsible for executing the challenge.

**Table 2:** Descriptive information on clinics participating in the Canadian Population Health Management Challenge.

Clinic type and clinic number		No. of practices	Location	Size^a^	Record system type	Role of participant
**EMR** ^b^ **-enabled clinics**						
	1	1	Ontario	7500	EMR	Office Manager
	2	1	Ontario	7400	EMR	Physician
	3	1	New Brunswick	8300	EMR	Physician
	4	1	Quebec	22,300	EMR	Physician
	5	3	Ontario	65,000	EMR + analytics database	Office Manager and IT^c^specialist
	6	4	Nova Scotia	4100	EMR	IT Director
	7	4	British Columbia	8500	EMR	Office Manager
	8	2	Ontario	150,000	EMR	Office Manager
**Paper-based clinics**						
	1	2	Quebec	27,800	Paper + eBilling + eAppointments	IT Manager
	2	1	Quebec	23,000	Paper	Archivist
	3	1	Newfoundland	3000	Paper	Physician

^a^Active patient = 1 count.

^b^Electronic medical record.

^c^Information technology.

**Figure 1:**
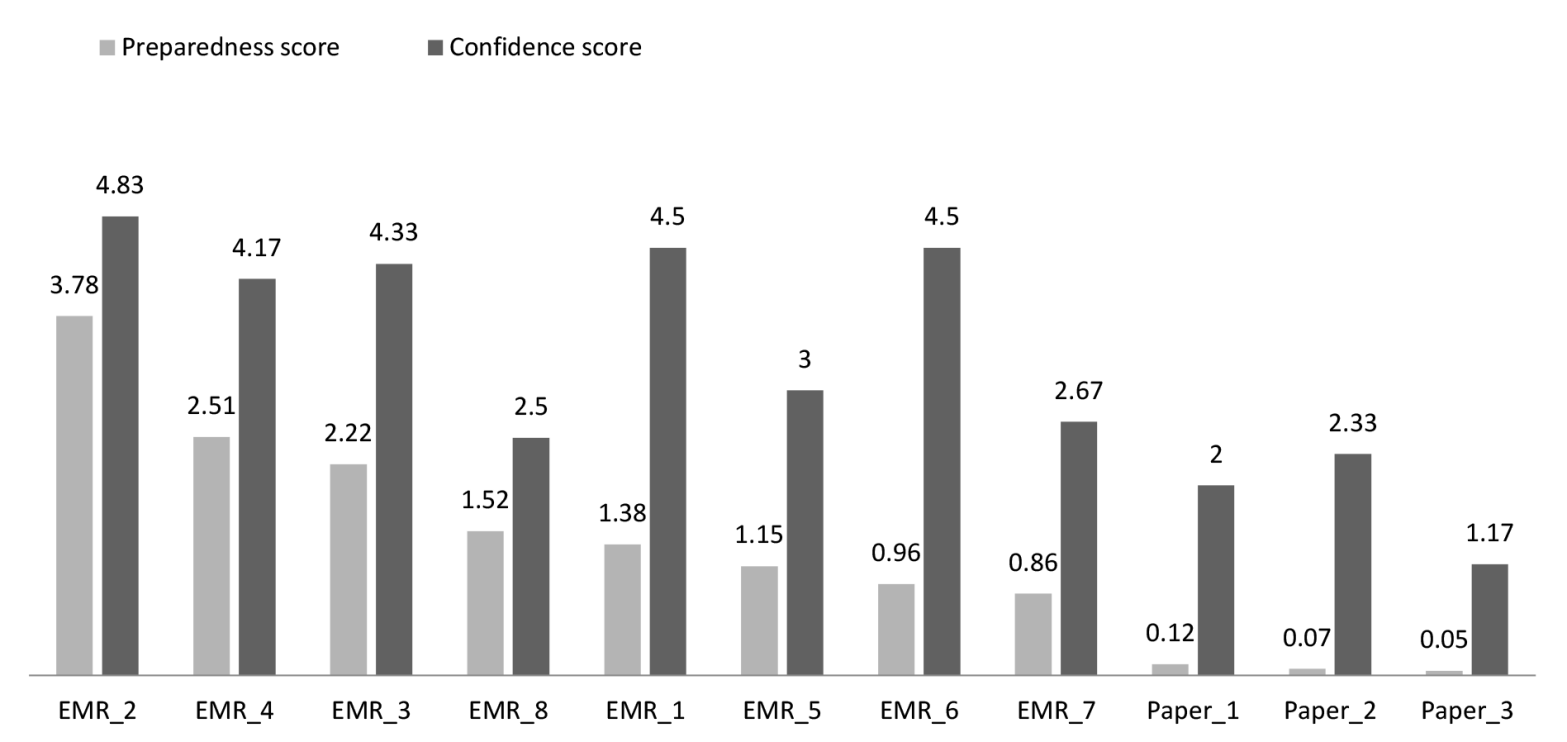
Overall ranking based on the average preparedness score across included modules in the Canadian Population Health Management Challenge. EMR: Electronic medical record-enabled clinics; Paper: Paper-based clinics.

Figure 1 illustrates each clinic’s rankings based on their overall average preparedness score. See Multimedia Appendix 2 for a detailed data summary of the module results for participating clinics.

Overall, EMR-enabled clinics completed a full review (100% of active patient records) in an average of 1.37 hours. Paper-based clinics reviewed approximately 10% of charts in 3.9 hours, thus requiring an estimated 40 hours (or 1 work week) to complete a full practice review (Multimedia Appendix 2). On a scale of 1 to 5, EMR-enabled clinics were more confident than paper-based clinics that they had captured all eligible patients (overall average 3.8 vs 1.9, respectively). Figure 1 illustrates the overall preparedness score and self-reported participant confidence in completion of reviews for paper-based and EMR-enabled clinics. While an expected capacity gap does exist between EMR-enabled and paper-based clinics (0.86–3.78 vs 0.05–0.12, respectively), results suggest a broad range among the EMR-enabled clinics, which may be due to a variety of factors to support or hinder capacity for PBPH.

To better understand the discrepancies between the clinics’ preparedness and performance on conducting the challenge, we relied on our qualitative data. The analysis helped clarify the main challenges and critical issues that facilitate PBPH in primary care settings.

Overall, our analysis showed that participants saw data retrieval as a critical activity in their current practice, but many mentioned that they do not perform it frequently enough or on a regular basis. Yet some indicated that data retrieval may not be equally important for every staff member or clinician depending on their role in patient and population health management.

Clinical teams tend to collect a lot of information in an EMR; all interviewees were concerned that this rich source of data was not always exploited adequately. The most common challenge found to inhibit proper use of data was the logistics of data retrieval. Most participants believed that data retrieval is a difficult, time-consuming task that was not comprehensive enough in their clinic, and has technical problems and limitations that influence database updates and integration. Exploiting the data (eg, writing queries, doing data analysis) was also regarded as a complex process that necessitates both a good understanding of the system’s functionalities and good access to the raw data. Almost all participants mentioned that they were not satisfied with their current data retrieval process in their practice, mostly because of the technical limitations of their systems and lack of resources required to keep track of and manage the quality of the data stored in their system.

In the participants’ opinion, the most important action to be taken to improve the data retrieval process was to standardize the data items, tools, and data entry forms. Most clinics emphasized the need for an easier-to-use and more consistent data entry method and codification, so that it would reduce the complexity for physicians. They also highlighted the need for making systems more user-friendly and easier to navigate for the average staff member or clinician, particularly for those without advanced computer or statistical programming knowledge or skills.

Overall, participants assessed their participation in the challenge as a positive experience, which helped them validate their views about EMRs. It also reinforced their ideas about the effectiveness and efficiency of their current work practices and systems used in the clinic, and that it highlighted limitations. For paper-based clinics, our results supported the importance of investing in EMRs, and displayed the significant differences in terms of processing efficiencies of PBPH. For EMR-enabled clinics, the study highlighted to management and staff the ways in which they can improve their use of the system (eg, investing in training and education initiatives for current and future clinicians, establishing EMR data standards, and developing data abstraction and presentation tools), as well as where they can enhance existing tools to improve work habits, quality of care, and performance.

## Discussion

Our study aimed to assess the capacity and preparedness of primary care settings for PBPH. First, we developed a preparedness score to reflect upon the relative performance of the participating clinics. The sampling approach allowed the performance of paper-based manual record systems to be compared to that of EMR-enabled or automated patient record systems.

While the preparedness score shows that EMR-enabled clinics have a higher capacity and confidence in PBPH reviews, our results also highlight a gap in the ranges of preparedness scores observed. We found a 7-fold (7.2 times) difference between the best-performing paper-based clinic and the worst-performing EMR clinic. Although a performance gap was to be expected based on existing research [14], the clinic scores showed that the actual gap is very significant. Our results also demonstrated a large gap (4.2 times) between the best-performing EMR clinic and the worst-performing EMR clinic. Based on our qualitative interviews and observation, we further found that this gap is mainly related to the absence of clear, user-friendly functional requirements regarding the use of and access to patient-level data within the current EMR systems being used by participating clinics.

In the cases of the best-performing EMR clinics, the challenge participant was a physician, as opposed to an archivist, medical office assistant, or information technology professional, who was reporting on behalf of a single practice. These clinics achieved a performance level that was, at least, 1.5 times better than the subsequent clinic among the ranked scores. Our qualitative data suggest that familiarity with the record layout and its content could explain the enhanced performance. Therefore, performance across all EMRs could be improved by incorporating data entry standards as well as coding standards. In this regard, data entry standards (eg, HL7 clinical document architecture) and coding standards (eg, SNOMED-CT) would allow medical personnel to “know where to look” and to effectively use the search capabilities of EMRs in support of PBPH. As discussed by participants, searching text fields for misspelled or aliased terms presents added complexity to the review and is also time consuming, which can further negatively affect the implementation of PBPH into practice workflow. Overall, these results are consistent with previous findings that emphasize the important role of EMRs (or information technology, in general) as the necessary factor in transforming the quality of primary care services [39,40].

Regarding paper-based clinics, the best-performing clinic achieved a performance level that was 1.7 times better than the next paper-based clinic and 2.4 times better than the lowest-level paper-based clinic. The observational data suggest that this enhanced performance was due to the mixed search strategy used by the best-performing clinic. In each module of the challenge, the initial population was determined using data held in the clinic’s electronic scheduling and electronic billing systems. Once these sources had provided a narrowly defined population list, the paper charts were reviewed. The repurposing of these electronic systems allowed this clinic to effectively cross-reference their patient records and establish initial subpopulations. From this, it can be seen that PBPH could possibly be undertaken by a paper-based clinic using a series of cross-reference tables or registries. This approach could prove effective in small practices where the administrative burden of maintaining the registries could be minimized. However, in a large practice, the strategy would be extremely labor intensive and subject to completeness concerns.

Most clinics chose to report on a single clinician’s practice. However, in a few cases, multiple practices were included. In line with the general findings, our data imply that, in these cases, EMR-enabled clinics have a clear advantage over paper-based clinics. In a paper-based clinic, the formulation of the query, which is the actual act of pulling a filed chart and looking at composite clinical notes and information, must be undertaken anew for each practice. In an EMR-enabled clinic, the formulation of the query is done for the first practice but is simply reused for the subsequent practice(s).

Finally, the analysis of follow-up interviews revealed the key challenges clinicians face in PBPH management. The most important issue in pursuing PBPH is the lack of systematic data storage retrieval practices in clinics. Despite advances in using information systems in health care contexts (whether for PBPH or not), many clinicians still perceive EMR use as an encounter-based electronic patient chart, instead of a tool to support prospective care and panel management [41]. Despite this, recent reports also highlight technical barriers in data retrieval and protection of privacy [42]. We also found that standardization of data and integration of databases are important steps in overcoming the challenges related to PBPH. These results are comparable with the results of previous studies that have emphasized the integration of databases and medical records that collect patient data from different sources or users [43].

### Limitations

We must acknowledge some limitations to this study. Neither the instrument of measure (the challenge) nor the measure derived from the instrument (the preparedness score) was rigorously validated. Validation of the preparedness score could prove advantageous, as it could be a tool for government agencies and clinic managers to evaluate the degree of preparedness and to assess the required effort and cost in undertaking PBPH. Nevertheless, to date, the preparedness score has exhibited important interpretation properties that would support the evaluation of the cost-benefit of different medical record keeping processes.

### Conclusion

The results of this study suggest that the PBPH preparedness score reflects the preparedness of the clinics participating in the challenge. The use of an EMR seems pivotal in setting the foundation to support PBPH management in primary care and subsequently to drive the associated beneficial outcomes for patients and clinicians. The range of capacity in EMR-enabled clinics suggests that for PBPH management to be effectively undertaken, key determinants of EMR optimization need to be addressed.

Despite the limitations, the study provides important contributions. The insights proposed by our findings can be used to show the criticality of EMR adoption for pursuing PBPH management. Although the results of previous studies looking at the advantages of EMR adoption have been mixed [22,25,26], our findings support the positive and significant effects of EMR use for improving the performance of clinics’ PBPH practice and the potential to affect quality of care and patient outcomes. The 2015 Commonwealth Fund survey of primary care physicians reports that EMR adoption has advanced substantially among Canadian and US primary care clinics (73% and 82%, respectively) [30]. However, use of multifunctionalities to support population health management remains below the international average [30]. This study adds to our existing knowledge of the potential benefits such systems can provide to primary health care providers and emphasizes the need for investing in initiatives to support current and future clinicians to overcome the challenges related to using data for proactive preventive and care management purposes. Furthermore, we established a tool (preparedness score) that provides a basis for comparing and contrasting the capacity of clinics to conduct evidence-informed PBPH management practices. Based on the score, stakeholders can understand the capacity of clinics’ preparedness to apply PBPH efforts in a clinical setting. Overall, using a similar challenge and preparedness score can shed light on the feasibility of population health management and the issues that should be addressed in order to implement it fully with associated resources in a specific context. Finally, policy makers and EMR vendors can use the qualitative findings to help regulate and improve future EMR systems, databases, and audit-reporting analytical functionalities. Creating easy-to-use EMR systems for clinical and care teams with a straightforward, optimized design, and retrieval and analytical features to support existing clinical practices and workflow, with integrated standards (especially with regard to data entry), is among the key factors to support advanced use of EMR data for quality outcomes of patient care through PBPH.

## Appendix

Multimedia Appendix 1 Sample screenshots of the Challenge.

Multimedia Appendix 2 Time and completeness ratio of modules for each clinic.

## Abbreviations

EMR: electronic medical record
EHR: electronic health record
PBPH: practice-based population health
