# Supplementary material for: Understanding the Impact of Electronic Medical Record Use on Practice-Based Population Health Management: A Mixed-Method Study
Source: JMIR Med Inform. 2016 Apr 4;4(2):e10. doi: 10.2196/medinform.4577 (PMC4835667; doi:10.2196/medinform.4577)
Supplement: Multimedia Appendix 1 [file medinform_v4i2e10_app1.pdf]

The Population Health Management Challenge

a research collaboration by: MEdbASE Research McGill

Site Information

Contact:

Clinic name:

Address:

City:

Province:

Country:

Postal code:

Telephone number:

web site:

Contact name:

Title:

Telephone number:

email address:

Description:

Provide brief description of services offered:

Number of physicians at site:

Number of physicians participating in the Challenge:

Number of GPs:

Number of Specialists:

Number of Nurse Practitioners:

Number of Allied Health Professionals:

(e.g. Nutritionist, Physiotherapist, etc)

Number of Medical Office Staff:

Total number of active patients*:

Please indicate how the active pat count was determined: Estimate

EMR: (electronic medical record)

Please select the type of medical records sytem in use at this site: Electronic medical records

How long has the particular EMR been in use at this site? Less than 6 months

Please indicate the degree to which the following technologies are used at this site:

	Yes, use routinely / Yes	Yes, use occasionally	No	Not sure
1 Electronic entry of clinical notes, including medical history and follow-up notes.				
2 Electronic entry of allergies **(using standardized coded fields; not free text)**.				

* a patient is active if he / she has visited the clinic or has been visited at home by a clinic physician in the last 5 years

Please indicate the degree to which the following technologies are used at this site:

		Yes, use routinely / Yes	Yes, use occasionally	No	Not sure
3	Electronic entry of immunizations **(using standardized coded fields; not free text)**.				
4	Electronic alerts or prompts about a potential problem with drug dose or drug interaction				
5	Generate lists of patients who are overdue for tests or preventative care				
6	Electronic interface to other external health care services or systems (e.g. hospitals, other clinics) for accessing or sharing patient information				
7	Electronic ordering of laboratory test results				
8	Electronic access to patient laboratory test results				
9	Electronic access to a comprehensive history of lab results (ordered by other physicians) for a patient				
10	Electronic preparation of a medication prescription				
11	Electronic transfer of prescriptions				
12	Electronic access to a comprehensive medication history (ordered by other physicians) for a patient				

The Population Health Management Challenge

a research collaboration by:

MEdbASE Research McGill

Practice Information

Physician:

Name: Physician 1

Specialty: Family practice

Gender:

Year of graduation:

Years practicing at Clinic:

Compensation model at Clinic: Fee for service

Description:

Total number of active patients*:

Please indicate how the active patient count was determined: Estimate

EMR: (electronic medical record)

Please select the type of medical records sytem in use at this site: Electronic medical records

How long has the particular EMR been in use at this site? Less than 6 months

Please indicate the degree to which the following technologies are used at this site:

	Yes, use routinely / Yes	Yes, use occasionally	No	Not sure
1 Electronic entry of clinical notes, including medical history and follow-up notes.				
2 Electronic entry of allergies **(using standardized coded fields; not free text)**.				
3 Electronic entry of immunizations **(using standardized coded fields; not free text)**.				
4 Electronic alerts or prompts about a potential problem with drug dose or drug interaction				

* a patient is active if he / she has visited the clinic or has been visited at home by a clinic physician in the last 5 years

Please indicate the degree to which the following technologies are used at this site:

		Yes, use routinely / Yes	Yes, use occasionally	No	Not sure
5	Generate lists of patients who are overdue for tests or preventative care	☐	☐	☐	
6	Electronic interface to other external health care services or systems (e.g. hospitals, other clinics) for accessing or sharing patient information	☐	☐	☐	
7	Electronic ordering of laboratory test results	☐	☐	☐	
8	Electronic access to patient laboratory test results	☐	☐	☐	
9	Electronic access to a comprehensive history of lab results (ordered by other physicians) for a patient	☐		☐	☐
10	Electronic preparation of a medication prescription	☐	☐	☐	
11	Electronic transfer of prescriptions	☐		☐	☐
12	Electronic access to a comprehensive medication history (ordered by other physicians) for a patient	☐		☐	☐

The Population Health Management Challenge

a research collaboration by: MEdbASE Research McGill

Challenge 1 (45 minutes)

Task:
Identify all active patients* over the age of 65 and indicate those that have not received vaccination against pneumococcal pneumonia in the last five years (prior to 2006).

Note:
The objective is to demonstrate the ability to produce a list of patients who require preventive care or tests.

Data report:

Number of patients aged 65 or older:		Please indicate how the patient count was determined:	Count from paper chart
Of the number above, indicate the number of patients that have not received an anti-pneumonia vaccine in the last five years (prior to 2006):		Please indicate how the non-vaccinated count was determined:	Count from paper chart
Of the number above, indicate the number of patients that refused the inoculation.		Please indicate how the refusal count was determined:	Count from paper chart

On a scale of 1 to 5, where 1 is not confident and 5 is very confident, please indicate how confident you are that the lists generated by this challenge can be used to contact all the eligible patients in your practice in an effort to have them participate in an inoculation clinic.

(●) 1 ( ) 2 ( ) 3 ( ) 4 ( ) 5

* a patient is active if he / she has visited the clinic or has been visited at home by a clinic physician in the last 5 years

The Population Health Management Challenge

a research collaboration by:

MEdbASE Research McGill

Challenge 2 (45 minutes)

Task:
Identify all active patients* who have suffered a myocardial infarct and indicate those for whom a 'statin' medication has not been prescribed.

Note:
The objective is to demonstrate the ability to produce a sub-list of patients based on a medical condition and recommended treatment.

Data report:

Number of patients having suffered a myocardial infarct: [ ] Please indicate how the patient count was determined: Count from paper chart

Of the number above, indicate the number of patients not prescribed a 'statin' medication (HMG CoA reductase inhibitor): [ ] Please indicate how the 'statin' count was determined: Count from paper chart

On a scale of 1 to 5, where 1 is not confident and 5 is very confident, please indicate how confident you are that the lists generated by this challenge can be used to contact all the eligible patients in your practice and to provide an appropriate prescription for each.

○ 1 ○ 2 ○ 3 ○ 4 ◉ 5

* a patient is active if he / she has visited the clinic or has been visited at home by a clinic physician in the last 5 years

The Population Health Management Challenge

a research collaboration by: MEdbASE Research McGill

Challenge 3 (60 minutes)

Task:
Prepare a registry, including phone numbers, of all active patients* who are female over the age of 50 and identify those that have not had a mammogram in the last three years.

Note:
The objective is to demonstrate the ability to produce actionable tasks through notification for patients and practice staff.

Data report:

Number of female patients 50 years of age or older:		Please indicate how the patient count was determined:	Count from paper chart
Of the number above, indicate the number of patients for whom the Clinic has a contact telephone number:		Please indicate how the telephone number count was determined:	Count from paper chart
Of this second count, indicate the number of patients that have not had a mammogram in the last three years:		Please indicate how the mammogram count was determined:	Count from paper chart
Of this last count, indicate the number of patients that have a prior diagnosis of breast cancer:		Please indicate how the breast cancer diagnosis count was determined:	Count from paper chart
Of the second count, indicate the number of patients that have a family history of breast cancer:		Please indicate how the family history count was determined:	Count from paper chart

On a scale of 1 to 5, where 1 is not confident and 5 is very confident, please indicate how confident you are that the lists generated by this challenge can be used to contact all the eligible patients in your practice and to provide an appropriate screening requisition for each.

- (•) 1
- ( ) 2
- ( ) 3
- ( ) 4
- ( ) 5

* a patient is active if he / she has visited the clinic or has been visited at home by a clinic physician in the last 5 years

The Population Health Management Challenge

a research collaboration by:

MEdbASE Research McGill

Challenge 4 (45 minutes)

Task:
Prepare a registry, including contact information, of all active patients* who are taking the drug 'metformin' and have a creatinine result greater than 150. With the registry in hand, assess the practice's ability to perform a recall of this medication.

Note:
The objective is to demonstrate the ability to provide relevant information to and possibly execute a drug recall based on a health authority's request.

Data report:

Number of patients taking 'metformin: [ ] Please indicate how the patient count was determined: Count from paper chart

Of the number above, indicate the number of patients having a creatinine greater than 150: [ ] Please indicate how the creatinine count was determined: Count from paper chart

Based on the resulting list of patients, could the practice contact each patient and indicate that they need to stop taking the medication and come in to see the doctor? [ ] Yes [ ] No Please indicate how the patients would be contacted: Staff contact by phone

In the next 48 hours, could the practice prepare a report for the Regional health Authority (or appropriate authority in your province) that details: [ ] Yes [ ] No Please indicate how the report would be prepared. Staff to compile and dra

1 The percentage of eligible patients contacted by the practice;
2 The percentage of eligible patients contacted directly who were successfully advised to discontinue use of the medication; and
3 The percentage of eligible patients successfully provided an appointment to see the physician.

On a scale of 1 to 5, where 1 is not confident and 5 is very confident, please indicate how confident you are that the lists generated by this challenge can be used to contact all the eligible patients in your practice and execute the drug recall.

(●) 1 ( ) 2 ( ) 3 ( ) 4 ( ) 5

* a patient is active if he / she has visited the clinic or has been visited at home by a clinic physician in the last 5 years

The Population Health Management Challenge

a research collaboration by:

MEdbASE Research McGill

Challenge 5 (60 minutes)

Task:
Identify all active patients* diagnosed with type 2 diabetes and indicate those for whom the latest HgbA1C test indicates a value greater than 0.070.

Note:
The objective is to demonstrate the ability to produce a metric that can be compared to national guidelines or peer comparison groups.

Data report:

Number of patients diagnosed with type 2 diabetes:		Please indicate how the patient count was determined:	Count from paper chart
Of the number above, indicate the number of patients with their most recent HgbA1C test indicating a value greater than 0.070:		Please indicate how the HgbA1C count was determined:	Count from paper chart
What is the percentage of type 2 diabetics with a HgbA1C greater than 0.070 in your practice that have received diabetes education and support?		Please indicate how this percentage was determined:	Calculated for this challe

On a scale of 1 to 5, where 1 is not confident and 5 is very confident, please indicate how confident you are that the lists generated by this challenge can be used to provide or develop comparative metrics for type 2 diabetics that would help in the identifcation of subpopulations requiring improved diabetes management.

(●) 1 ( ) 2 ( ) 3 ( ) 4 ( ) 5

* a patient is active if he / she has visited the clinic or has been visited at home by a clinic physician in the last 5 years

The Population Health Management Challenge

a research collaboration by:

MEdbASE Research McGill

Challenge 6 (45 minutes)

Task:
Prepare a registry, including contact information, of all active patients* who are taking the drug 'Avandia' and are suffering from congestive heart failure (CHF).

Note:
The objective is to demonstrate the ability to provide relevant information in support of a drug recall based on a health authority's request.

Data report:

Data report:

Number of patients taking 'Avandia':		Please indicate how the patient count was	Count from paper chart
Of the number above, indicate the number of patients exhibiting a propensity for CHF flare-ups:		Please indicate how the CHF count was determined:	Count from paper chart

On a scale of 1 to 5, where 1 is not confident and 5 is very confident, please indicate how confident you are that the lists generated by this challenge can be used to contact all the eligible patients in your practice and execute the drug recall.

(●) 1 ( ) 2 ( ) 3 ( ) 4 ( ) 5

* a patient is active if he / she has visited the clinic or has been visited at home by a clinic physician in the last 5 years
