# Supplementary material for: Understanding the Impact of Electronic Medical Record Use on Practice-Based Population Health Management: A Mixed-Method Study
Source: JMIR Med Inform. 2016 Apr 4;4(2):e10. doi: 10.2196/medinform.4577 (PMC4835667; doi:10.2196/medinform.4577)
Supplement: Multimedia Appendix 2 [file medinform_v4i2e10_app2.pdf]

Multimedia Appendix 2. Time and completeness ratio of modules for each clinic

Clinic	Module 1				Module 2				Module 3			
	Time (Min)	%	confidence	preparedness	Time (Min)	%	confidence	preparedness	Time (Min)	%	confidence	preparedness
EMR_1	1	100	5	N/C	48	100	4	0.94	50	100	4	1.2
EMR_2	20	100	5	2.25	17	100	4	2.65	15	100	5	4
EMR_3	1	100	3	N/C	20	100	5	2.25	28	100	4	2.14
EMR_4	40	100	1	0.47	35	100	5	1.29	57	100	4	0.51
EMR_5	3	100	3	N/C	48	100	1	0	30	100	5	1.2
EMR_6	30	100	5	0.78	46	100	3	0.24	48	100	4	0.58
EMR_7	83	10	3	0.28	64	100	3	0.62	58	100	3	0.62
EMR_8	51	100	3	0.7	28	100	5	1.61	86	100	3	0.68
Paper_1	44	10	2	0.1	40	10	2	0.11	44	10	2	0.14
Paper_2	40	7	2	0.08	44	7	2	0.07	58	7	3	0.07
Paper_3	32	4	1	0.06	24	4	1	0.08	32	4	1	0.02
Clinic	Module 4				Module 5				Module 6			
	Time (Min)	%	confidence	preparedness	Time (Min)	%	confidence	preparedness	Time (Min)	%	confidence	preparedness
EMR_1	19	100	5	2.37	60	100	4	1	24	100	5	1.88
EMR_2	13	100	5	3.46	12	100	5	5	5	100	5	9
EMR_3	15	100	5	3	40	100	4	1.5	0	100	5	N/C
EMR_4	13	100	5	3.46	13	100	5	4.62	0	100	5	N/C
EMR_5	34	100	5	1.32	29	100	2	2.07	6	100	2	7.5
EMR_6	28	100	5	0.6	8	100	5	2.5	0	100	5	N/C
EMR_7	15	100	3	1.5	32	100	2	0.63	1	100	2	N/C
EMR_8	35	100	1	1.29	24	100	1	2.5	2	100	2	N/C
Paper_1	44	10	2	0.1	57	10	2	0.11	31	10	2	0.15
Paper_2	45	7	3	0.07	64	7	2	0.07	46	7	2	0.07
Paper_3	46	4	1	0.04	40	4	1	0.06	30	4	2	0.08

Note: All Confidence values are in a rage of 1 to 5, where 1 is not confident and 5 is very confident that the information is actionable.

The preparedness score shows the capacity to conduct PBPH management.
